# *In vitro* versus *in situ* biofilms for evaluating the antimicrobial effectiveness of herbal mouthrinses

**DOI:** 10.3389/fcimb.2023.1130255

**Published:** 2023-01-31

**Authors:** Nicole Schönbächler, Thomas Thurnheer, Pune Nina Paqué, Thomas Attin, Lamprini Karygianni

**Affiliations:** ^1^ Clinic of Conservative and Preventive Dentistry, Center of Dental Medicine, University of Zurich, Zurich, Switzerland; ^2^ Clinic of Reconstructive Dentistry, Center of Dental Medicine, University of Zurich, Zurich, Switzerland

**Keywords:** multispecies oral biofilm, chlorhexidine (CHX), confocal laser scanning microscopy (CLSM), herbal mouthrinses, *in situ*

## Abstract

For centuries, diverse mouthrinses have been applied for medicinal purposes in the oral cavity. In view of the growing resistance of oral microorganisms against conventional antimicrobial agents e.g. chlorhexidine, the implementation of alternative treatments inspired by nature has lately gained increasing interest. The aim of the present study was to compare *in vitro* biofilm models with *in situ* biofilms in order to evaluate the antimicrobial potential of different natural mouthrinses. For the *in vitro* study a six-species supragingival biofilm model containing *A. oris*, *V. dispar*, *C. albicans*, *F. nucleatum*, *S. mutans* and *S. oralis* was used. Biofilms were grown anaerobically on hydroxyapatite discs and treated with natural mouthrinses Ratanhia, Trybol and Tebodont. 0.9% NaCl and 10% ethanol served as negative controls, while 0.2% CHX served as positive control. After 64h hours, biofilms were harvested and quantified by cultural analysis CFU. For the *in situ* study, individual test splints were manufactured for the participants. After 2h and 72h the biofilm-covered samples were removed and treated with the mouthrinses and controls mentioned above. The biofilms were quantified by CFU and stained for vitality under the confocal laser scanning microscope. In the *in vitro* study, 0.2% CHX yielded the highest antimicrobial effect. Among all mouthrinses, Tebodont (4.708 ± 1.294 log10 CFU, median 5.279, p<0.0001) compared with 0.9% NaCl showed the highest antimicrobial potential. After 72h there was no significant reduction in CFU after 0.2% CHX treatment. Only Trybol showed a statistically significant reduction of aerobic growth of microorganisms *in situ* (5.331 ± 0.7350 log10 CFU, median 5.579, p<0.0209). After treatment with the positive control 0.2% CHX, a significant percentage of non-vital bacteria (42.006 ± 12.173 log10 CFU, median 42.150) was detected. To sum up, a less pronounced effect of all mouthrinses was shown for the *in situ* biofilms compared to the *in vitro* biofilms.

## 1 Introduction

For centuries, diverse mouthrinses have been applied for medicinal purposes in the oral cavity. To date, numerous chemical ingredients and natural compounds, which are able to eliminate biofilms, have been subjects of current scientific research as potent ingredients of oral mouthrinses (Milleman et al., 2022). The reason for the increasing scientific interest is that biofilms cause serious infections in different parts of the human body and, especially in the oral cavity such as caries, periodontal disease or peri-implantitis (Spuldaro et al., 2021). Until now, several antimicrobial substances have been tested for the control of oral biofilms, including but not limited to chlorhexidine (CHX), essential oils, amine fluoride or triclosan. For over 40 years, CHX has been known as an excellent mouthrinse to control dental plaque and thereby prevent gingival inflammation (Cai et al., 2020). CHX is a cationic biguanide, which is bacteriostatic in low and bactericidal in higher concentrations is regarded as the gold standard (Abouassi et al., 2014). Yet, the use of CHX is associated with some well-known side effects, namely the reduction of human taste perception and discoloration of the tongue, composite fillings and teeth, which interfere with its application (Gürgan et al., 2006). It is also known that the antimicrobial activity of CHX is affected by the environment, also by the presence of organic compounds and food rests in the oral cavity (James et al., 2017). It is believed that the turnover rate of specific salivary proteins can decrease the activity of different agents with antimicrobial activity (Auschill et al., 2001; Auschill et al., 2005). In view of the growing resistance of oral microorganisms against conventional antimicrobial agents e.g. CHX, the implementation of alternative treatments inspired by nature has lately gained increasing interest (Karygianni et al., 2015; Shen et al., 2016; Cieplik et al., 2019). Due to the lack of secondary effects and a higher potential for long-term usage in the oral cavity, several natural plant extracts and plant-derived pure substances has been screened for the control of oral infections in recent studies (Karygianni et al., 2014; Tiwari et al., 2015; Cai et al., 2020; Kurz et al., 2021). Even the World Health Organization (WHO) published in 2014 recommendations to emphasize the importance of traditional and alternative phytomedicine for the well-being and presented many proposals to establish plant-based medicine (Kurz et al., 2021). In a systematic review on herbal interventions in the oral cavity was shown, that plant extracts as *Vitis vinifera*, *Camellia sinensis* or manuka honey exhibit high elimination rates of multispecies oral biofilms (Karygianni et al., 2015).

The oral cavity consisting of up to 700 different bacterial species is an enormously complex habitat, which is unique in the human body. The microbial cells, which are embedded in a matrix of extracellular polymeric substances, are irreversibly attached to epithelial- and tooth surfaces. Despite the dominance of adverse conditions (alternating temperatures, pH as well as oxygen and nutrient supplies) in the microenvironment of the oral cavity, biofilms survive thanks to commensal or mutualistic symbiotic relationships among different microbial species, allowing for the harmonic growth of both aerobic and anaerobic microorganisms (Wood et al., 2000). This physiochemical intercommunication pattern among oral microorganisms within a biofilm, can lead to extremely resistant biofilms, which can be up to 1000 times less susceptible to diverse antimicrobials when compared to planktonic microorganisms (Karygianni et al., 2014). Additionally, the heterogeneity and structural complexity of oral biofilms poses a great challenge for their treatment, which has been confirmed by the use of different biofilm models in several *in vitro* studies (Guggenheim et al., 2001). One of the most established models is a multispecies supragingival biofilm model, which consists of six different microbial species, was firstly established in Zurich and is widely known as “Zurich biofilm model” (Shapiro et al., 2002). The initial *in vitro* screening of potential antimicrobial substances is an important step for the selection of the agents to be applied in clinical trials. In such *in vitro* studies, several antimicrobial agents such as chlorhexidine (CHX), amine fluoride/stannous fluoride, triclosan and phenolic agents were shown to be effective against oral biofilms (Auschill et al., 2005). Furthermore, some clinical studies (Auschill et al., 2001; Auschill et al., 2005), examined the impact of antimicrobials on intact oral biofilms, suggesting that the antimicrobial agent-induced alterations within oral biofilms might considerably differ between *in situ* biofilms and *in vitro* biofilm models (Auschill et al., 2005). The aim of the present study was to compare *in vitro* biofilm models with *in situ* biofilms in order to evaluate the antimicrobial potential of different natural mouthrinses. The null hypothesis of the study was that *in vitro* and *in situ* biofilms yield comparable outcomes in terms of screening of potential plant-derived antimicrobial agents. The microbial growth was therefore quantified for the treated biofilms, which were visualized under the confocal laser scanning microscopy (CLSM) and finally analyzed using an image analysis software.

## 2 Materials and methods

### 2.1 *In vitro* biofilms

#### 2.1.1 Strains and preparation of inoculum for *in vitro* biofilms

For this study, the following six representative supragingival microorganisms were used: *Veillonella dispar* (OMZ 493), *Fusobacterium nucleatum* (OMZ 598), *Streptococcus oralis* (OMZ 607), *Streptococcus mutans* (OMZ 918), *Actinomyces oris* (OMZ 745) and *Candida albicans* (OMZ 110) The microorganisms were obtained by cultivation on Columbia blood agar plates (CBA; Becton, Dickinsson and Company, Sparks, MD, USA) supplemented with 5% (v/v) hemolyzed human blood under anaerobic conditions at 37°C. For overnight cultures, the microorganisms were picked from the CBA plates and inoculated into 9 ml filter-sterilized fluid universal medium + 0.3% glucose supplemented with 67 mmol/L Sørensen’s buffer, pH 7.2 (“modified fluid universal medium”, mFUM). As nutrition for *V. dispar*, 0.1% (v/v) sodium lactate was added into mFUM (Guggenheim et al., 2001). The cultures were then incubated anaerobically at 37°C for 15 h. 500 µl of the grown cultures were transferred into 5 ml preequilibrated mFUM, whereas the strains were again incubated for 5 h anaerobically at 37°C. After 5 h, the optical density (OD_550_) was measured for each strain and adjusted with fresh mFUM at 1.0 +/- 0.05. To obtain a microbial suspension the density-adjusted culture was mixed in equal volume.

#### 2.1.2 Collection of saliva

The saliva was collected in the morning between 9:00 and 10:00 o’clock, at least 1.5 h after eating, drinking or plaque control. Whole, unstimulated saliva was collected in sterile 50 ml tubes. Afterwards the collected saliva was pooled and centrifuged (30 min, 4°C, 27’500 x g). The supernatant was pasteurized (30 min, 60°C) and again centrifuged (30 min, 4°C, 27’500 x g). The supernatant was pipetted into 50 ml sterile tubes and stored at -20°C. For pellicle formation the hydroxyapatite discs (HA; Ø 9mm, Clarkson Chromatography Products, South Williamsport, PA, USA) were placed in 24-well polystyrene cell culture plates and covered with 800 µl of processed whole unstimulated pooled saliva from individual donors (Guggenheim et al., 2001).

#### 2.1.3 *In vitro* biofilm formation

Biofilms were grown in 24-well polystyrene cell culture plates on pellicle coated HA discs for 64 h under anaerobic conditions at 37°C to initiate the biofilm formation. For the first 16 h, 1120 µl of saliva (diluted 1:2 with 0.25% NaCl), 480 µl mFum (Fum + 0.3% glucose) and 200 µl of the prepared microbial suspension were added to the preconditioned pellicle-coated discs. The growth medium consisting of diluted saliva and mFUM with a carbohydrate concentration of 0.15% glucose and 0.15% sucrose (w/v) instead of 0.3% glucose was renewed after 16 h and 40 h, respectively. The 1-minute treatments with three different mouthrinses (**Table 1**) and control solutions 0.9% NaCl (negative control), 10% EtOH (negative control) and 0.2% CHX (positive control) were conducted 3 times a day for 2 days: after 16 h, 20 h, 24 h, 40 h, 44 h and 48 h. After treatment, the discs were washed twice in 3 x 2 ml physiological saline (0.9% NaCl), transferred back to the growth medium and incubated anaerobically at 37°C.

**Table 1:** Overview of the tested herbal mouthrinses, their active agents and the respective manufacturers.

Mouthrinses	Active ingredients	Manufacturer
Tebodont	*Melaleuca alternifolia*, sodium fluoride	Dr. Wild & Co. AG, Muttenz, Switzerland
Trybol	Chamomile, salvia, arnica	Trybol AG, Neuhausen, Switzerland
Rathania	Myrrhe, Ratanhia root, *Aesculus hippicastanum*, alcohol 57,6% diluted to 10% alcohol	Weleda, Schwäbisch Gmünd, Germany

#### 2.1.4 Natural mouthrinses

**Table 1** summarizes the tested mouthrinses along with their active agents. Tebodont (Dr. Wild & Co. AG, Muttenz, Switzerland), Ratanhia (Weleda, Schwäbisch Gmünd, Germany) and Trybol AG, Neuhausen, Switzerland) were tested. To evaluate the inhibitory effect of commercial mouthrinses on *in vitro* and *in situ* supragingival biofilms, Ratanhia and Trybol were diluted as recommended in the application instructions. Ratanhia was diluted to obtain final alcohol concentration of 10%, while Trybol was diluted with sterile water (1:20). All three of the chosen antimicrobial agents consist mainly of essential oils, as specified in **Table 1**. For the negative control, physiological saline (0.9% NaCl) and 10% ethanol were used. The inhibitory effect of 0.2% chlorhexidine (CHX) served as positive control.

#### 2.1.5 Biofilm harvesting

After 64 h the biofilms were rinsed three times in 0.9% NaCl to remove the non-adherent microorganisms. To harvest the biofilms, discs were transferred into 1 ml 0.9% NaCl, vortexed for 2 min and then sonicated for 5 s at 30 W (Sonifier B-12, Branson Ultrasonic, Urdorf, Switzerland). The resulting microbial suspensions were diluted in 0.9% NaCl by serial dilutions and plated on selective (Biggy Agar, Becton, Dickinsson and Company, Sparks, MD, USA) and non-selective agar plates (CBA) to assess the colony forming units (CFU). The plates were incubated for 72h under both aerobic (10% CO_2_) and anaerobic conditions.

### 2.2 *In situ* biofilms

#### 2.2.1 Subjects and specimens

Six volunteers from 25 to 59 years of age were selected. Prerequisites for participation in the study included (i) no use of antibiotics and mouthrinses three months prior to the wearing, (ii) no pregnancy or breastfeeding, (iii) no systemic diseases, and in addition, (iv) no oral hygiene was carried out in the 2 hours before wearing the appliance, (v) the consumption of food and liquids, as well as alcohol and nicotine, was prohibited during wearing the appliances and finally, (vi) the subjects had not participated in any other clinical examination up to 30 days before the start of the study. The written consent of the test persons to participate was given. The study was approved by the Ethics Committee of the University of Zurich (Basec-Number 2019-01324). After screening the patients’ dental health, the DMFT scores retrieved were between 0 and 13.

#### 2.2.2 Study design

**Figure 1:**
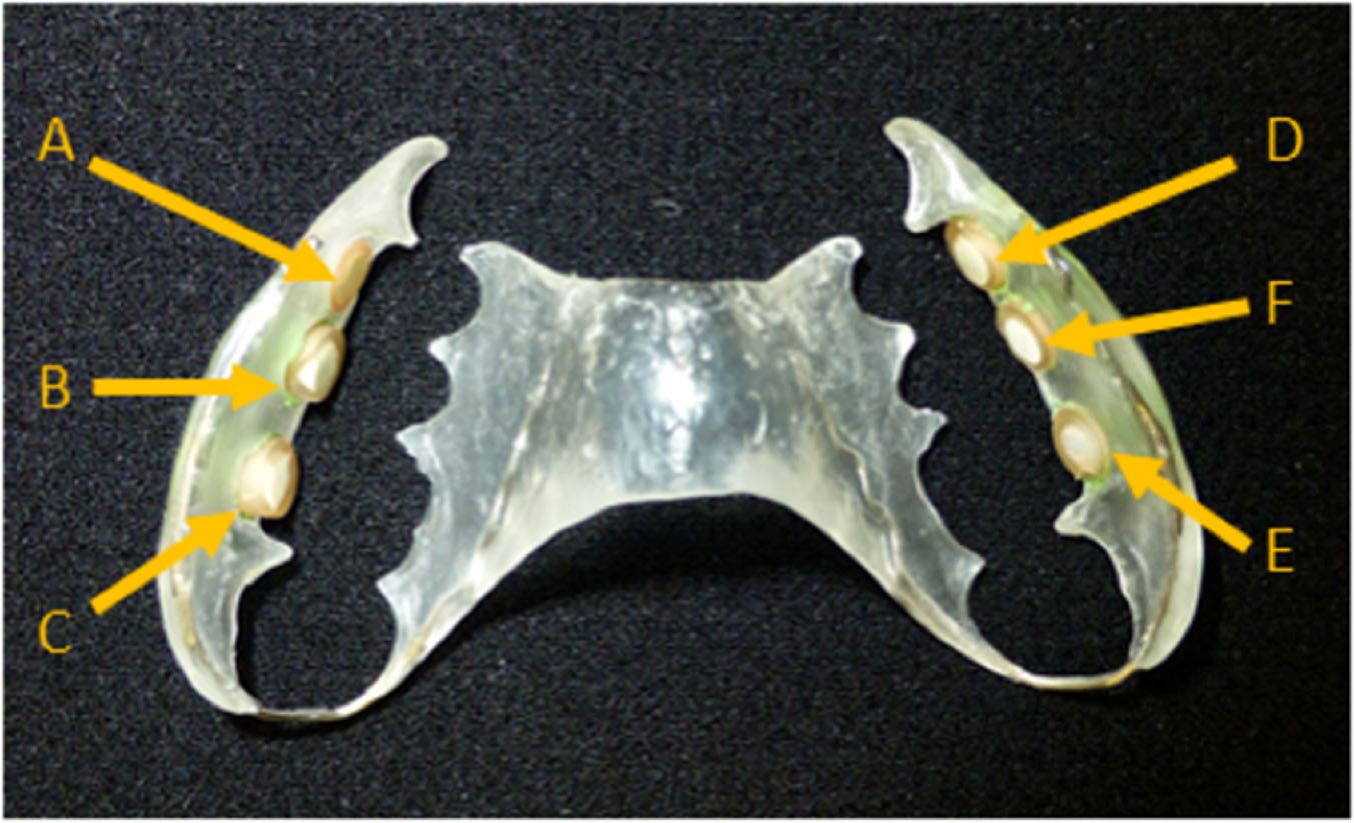
Individual acrylic splints with six enamel slabs attached to different locations. The slabs were placed in the front, in the middle and in the back on both sides, right and left. All sides of the slabs, except the enamel surface were covered with silicone and embedded in the appliance. Due to the fact that biofilm formation can differ from molar to premolar, the treatment groups were shifted one position further at each subject. C/E: Back; B/F: Middle; A/D: Front.

The bovine enamel slabs (BES, d = 6 mm, h = 3 mm) were gained from freshly extracted bovine spongiform encephalopathy-free teeth. For each volunteer an individual, intraoral splint system was constructed for the upper jaw **Figures 1**. Six bovine enamel slabs were fixed towards the molar and premolar region with an A-silicon compound (President plus light body, Coltène/Whaledent AG, Altstätten, Switzerland). To avoid disturbing tongue and cheek movements the acrylic splint was rounded at the end and the BES margins were covered with silicon. During treatments the maxillary splint was not allowed to be brushed. During meals and oral hygiene, the splint system was stored in physiological saline for a maximum of 1 hour and then rinsed only with water before being put back into the oral cavity. Following the removal of the splint system after the given test periods of 2 h or 3 days, they were stored in 0.9% NaCl. Subsequently, the biofilms were treated and harvested for determining the microbial growth (CFU) or stained for assessing cell viability using confocal laser scanning microscopy (CLSM), respectively.

### 2.3 Determination of colony forming units

The colony-forming units (CFUs) for 2h and 72h were evaluated quantitatively. The patients had to wear the splint system first for 1 min, then the BES were treated for 1 min with the mouthrinse and control solutions respectively. After that the discs were rinsed three times in 1 ml 0.9% NaCl. Subsequently, the BES were worn for 2h and 72h, respectively. After the removal of the splints from the oral cavity, the silicon was detached from the samples and the discs were then treated for 2 min with the mouthrinses and again dipped in 1 ml NaCl three times. Afterwards the discs were transferred in 1 ml 0.9% NaCl. To harvest the biofilms, the BES were vortexed for 30 sec, sonicated for 2 min and again vortexed for another 30 sec. From the resulting microbial suspensions, serial dilutions were done and plated on CBA. The plates were incubated for 72h under aerobic (10% CO_2_) and anaerobic conditions.

### 2.4 Biofilm staining

The obtained biofilms on the BES after 72 h were stained using nucleic acid stains. Following dyes from Invitrogen™ (ThermoFisher Scienfific, Reinach, Switzerland) were used for viability analysis and visualization of extracellular DNA (eDNA): SYTO 40 a cell-permeant nucleic acid stain, DRAQ7 and TOTO-1 cell impermeable DNA dyes. The BES were stained for 15 min at room temperature in the dark with a final concentration of 20 µM for SYTO 40 and DRAQ7 and 2 µM for TOTO-1. After staining, the discs were washed with H_2_O and then placed on upside down on chamber slides with a drop of Mowiol (Roth AG, Arlesheim, Switzerland).

### 2.5 Confocal laser scanning microscopy and image analysis

Subsequently, the stained biofilms were analyzed by confocal laser scanning microscopy using a Leica TCS SP5 microscope (Leica Microsystem, Wetzlar, Germany) with x 63/1.4 NA oil-immersion objective lens. Excitation of the dyes were carried out using the following wavelengths: UV laser 405 nm, Argon laser 488 nm and Helium-Neon laser 633 nm. Fluorescence emission was detected at the following wavelengths: 420-460 nm (SYTO 40), 515-570 nm (TOTO-1), and 687-770 nm (DRAQ7). The biofilms were scanned in sequential mode, and z-series were generated by vertical optical sectioning using a step size of 0.5 µm. Image acquisition was conducted in 6x line average mode. Each disc was measured at three different positions. Images were processed using IMARIS software (version 9.7.2, Bitplane, Zurich, Switzerland) and quantified by Fiji. The 3D data were collapsed into 2D by maximal z-projection and each channel was analyzed separately. Contrary to TOTO-1, the threshold of SYTO 40 and DRAQ7 stains was not modified. The measurements were set areas, min/max, gray value, area fraction, mean gray value and stack position and the size of the analyze particles were from 0.10 - infinity micron^2^.

### 2.6 Statistical analysis

For the *in vitro* study three independent experiments were conducted and within each experiment every treatment group was represented in triplicate (n=9). For the *in situ* study independent experiments of 6 subjects were carried out and within each subject every treatment group was represented once. The data points of the treatment groups were summarized of all subjects (n=6). For the quantification of the cell viability, 18 data points were generated, as each biofilm sample was scanned at 3 positions. Two-way analysis of variance (ANOVA) was used to analyze the difference in microbial growth per biofilm between the control groups (CHX, ethanol, NaCl) and the different treatments with herbal mouthrinses. For correction Tukey’s multiple comparisons test was used. Missing values were ascribed the lowest detection limit value of the assay to allow for logarithmic transformation. Statistics have been implemented using GraphPad Prism software (version 7; La Jolla, CA, USA). Significance level was set at p < 0.05.

## 3 Results

### 3.1 *In vitro* effects of mouthrinses

**Figure 2:**
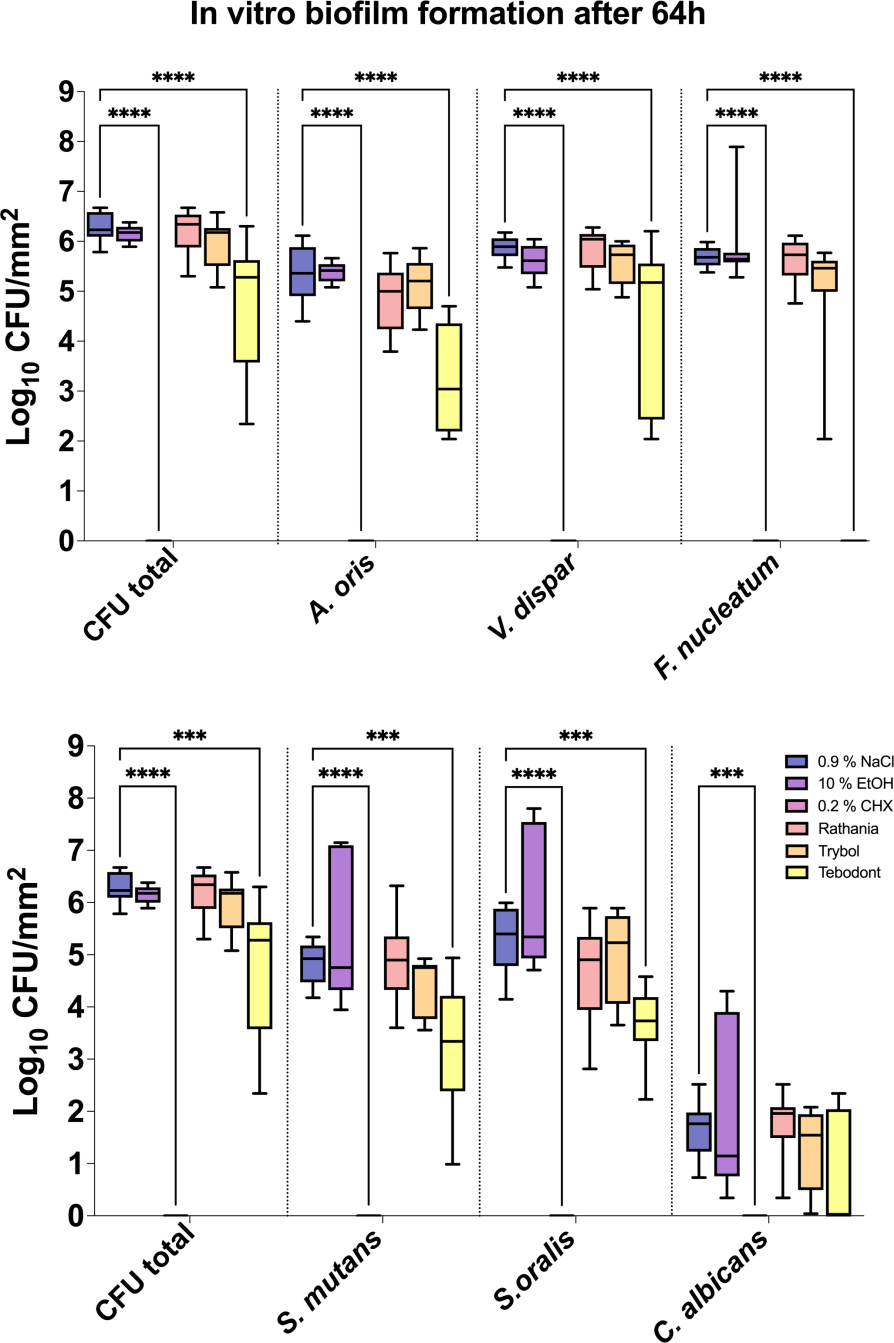
Box plots illustrating the colony-forming units (CFUs) of six-species oral biofilms after simultaneous exposure to the natural mouthrinses Ratanhia, Trybol, Tebodont. 0.2% CHX (positive control), 0.9% NaCl and 10% EtOH (negative control) were also used. The CFU values were shown on a log_10_ scale per mm^2^ (log_10_/mm^2^). The line dividing the box shows the median. The p-values of the significantly different data are plotted: ***= P-value <0.001, **** = P-value < 0.0001.

The antimicrobial effect of the tested mouthrinses on the microbial growth of supragingival biofilms consisting of six species grown for 64h *in vitro* is illustrated In **Figure 2**. The application of 0.2% CHX as positive control shows a significant decrease (0.000 ± 0.000 log_10_ CFU, median 0.000, *p< 0.0001*) on both aerobic and anaerobic microorganisms compared to the two negative controls (0.9% NaCl; 6.289 ± 0.289 log_10_ CFU, median 6.230, *p< 0.0001* and 10% ethanol; 6.154 ± 0.170 log_10_ CFU, median 6.176, *p< 0.0001*). A significant antimicrobial effect was also found for Tebodont (4.708 ± 1.294 log_10_ CFU, median 5.279, *p< 0.0001*) compared to the negative controls (0.9% NaCl and 10% EtOH). In comparison with Trybol (5.971 ± 0.498 log_10_ CFU, median 6.176, *p< 0.0138*) and Ratanhia (6.208 ± 0.454 log_10_ CFU, median 6.342, *p< 0.0016*), Tebodont presented a significant antimicrobial activity. Treatment with Ratanhia (6.208 ± 0.454 log_10_ CFU, median 6.342, *p> 0.9999*) or Trybol (5.971 ± 0.498 log_10_ CFU, median 6.176, *p> 0.9968*) failed to yield significant reduction of log_10_ CFU compared to the negative controls.

Furthermore, Tebodont induced a significant decrease of the bacterial counts of *A. oris* (3.260 ± 1.092 log_10_ CFU, median 3.041, *p> 0.0001*), *V. dispar* (4.342 ± 1.612 log_10_ CFU, median 5.176, *p> 0.0001*) and *F. nucleatum* (0.107 ± 0.319 log_10_ CFU, median 0.000, *p> 0.0001*). A substantial antibacterial effect of Tebodont was observed for *S. mutans* (3.239 ± 1.217 log_10_ CFU, median 3.342, *p> 0.0328*) and *S. oralis* (3.682 ± 0.683 log_10_ CFU, median 3.732, *p> 0.0011*). The microbial counts of *Candida albicans* solely *(*0.714 ± 1.074 log_10_ CFU, median 0.000, *p> 0.2653*) were not affected by the treatment with the herbal mouthrinses. Treatment with 0.2% CHX ended to a significant decrease of microbial counts of all six supragingival species (0.000 ± 0.000 log_10_ CFU, median 0.000, *p< 0.0001*).

### 3.2 *In situ* effects of mouthrinses

**Figure 3:**
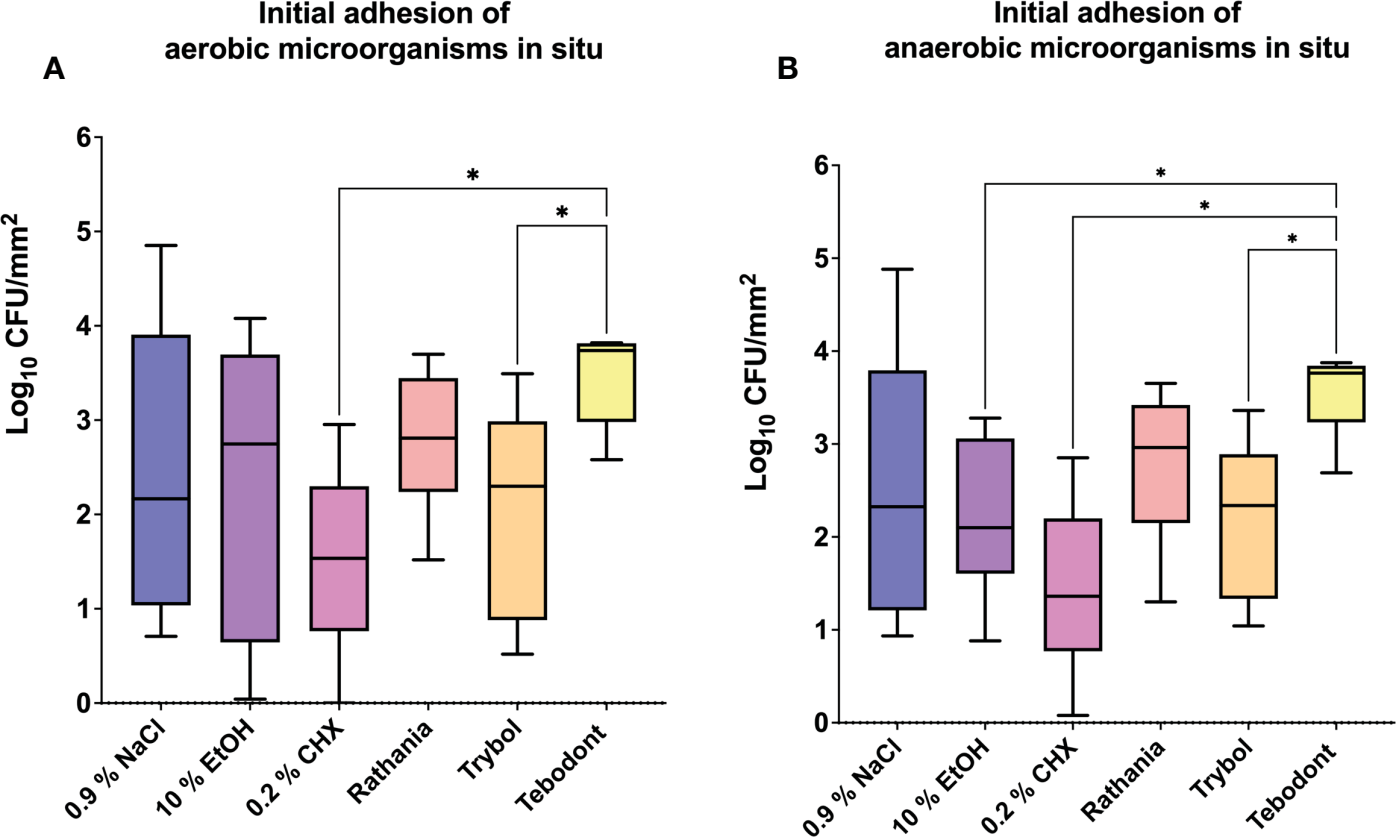
Box plots show the number of CFUs that demonstrate the antimicrobial effect of the tested substances on aerobic **(A)** and anaerobic **(B)** bacteria after an oral exposure time of 2 hours. A positive control (0.2% CHX), two negative controls (0.9% NaCl, 10% EtOH) and the natural mouthrinses Ratanhia, Trybol and Tebodont were also tested. The CFU values were shown on a log_10_ scale per mm^2^ (log_10_/mm^2^). The horizontal line within the box shows the median values. The p-values of the significantly different data are plotted: *= P-value <0.05.

The eradication rates of initially adherent oral aerobic and anaerobic microorganisms following treatment of *in situ* biofilms with different herbal mouthrinses are shown in **Figure 3**. Treatment with 0.2% CHX turned out to be very effective against the initial adhesion of aerobic (1.439 ± 1.219 log_10_ CFU, median 1.534) and anaerobic (anaerobic; 1.439 ± 0.9357 log_10_ CFU, median 1.361) microorganisms after 2h. In contrast, Tebodont, showed no killing effect *in situ* (aerobic; 3.467 ± 0.5111 log_10_ CFU, median 3.739, *p< 0.0336* and anaerobic; 3.557 ± 0.4561 log_10_ CFU, median 3.764, *p< 0.0248*). Contrary to Tebodont, a significant reduction of the microbial counts was detected for Trybol under both aerobic (2.071 ± 1.142 log_10_ CFU, median 2.298, *p< 0.0308*) and anaerobic conditions (2.207 ± 0.8791 log_10_ CFU, median 2.338, *p< 0.0431*).

**Figure 4:**
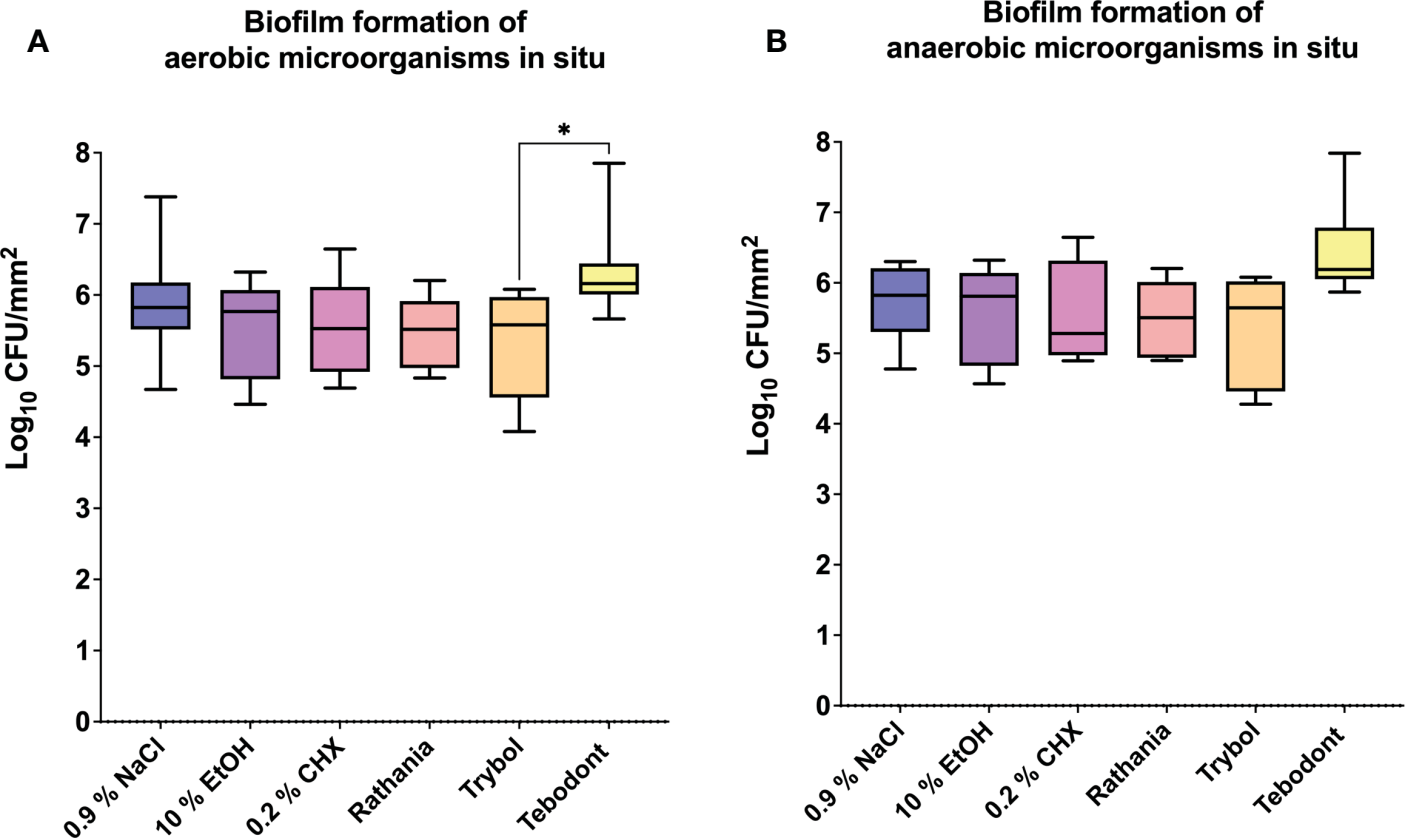
The graphs show the number of CFUs that demonstrate the antimicrobial effect of the tested substances on aerobic **(A)** and anaerobic **(B)** bacteria after an oral exposure time of 72 hours. A positive control (0.2% CHX), two negative controls (0.9% NaCl, 10% EtOH) and three different natural mouthrinses Ratanhia, Trybol and Tebodont were also used. The CFU values were shown on a log_10_ scale per mm^2^ (log_10_/mm^2^). The horizontal line within the box shows the median values. The p-values of the significantly different data are plotted: *= P-value <0.05.

Regarding the adherent oral microorganisms after 72h *in situ* (**Figure 4**), there was no statistically significant reduction of CFUs (log_10_/mm^2^) after treatment with 0.2 CHX (aerobic 5.525 ± 0.6452 log_10_ CFU, median 5.525, *p> 0.9999* and anaerobic 5.549 ± 0.7243 log_10_ CFU, median 5.280, *p> 0.9999*). In comparison to Tebodont, only Trybol showed a statistically significant difference in CFU (log_10_/mm^2^) of aerobic microorganisms *in situ* (5.331 ± 0.7350 log_10_ CFU, median 5.579, *p< 0.0209*). Interestingly, there was no significant difference between the negative controls 0.9% NaCl (aerobic 2.438 ± 1.562 log_10_ CFU, median 2.166, *p< 0.7711* and anaerobic 2.553 ± 1.478 log_10_ CFU, median 2.325, *p> 0.9999*),10% ethanol (aerobic 2.338 ± 1.629 log_10_ CFU, median 2.747, *p< 0.7205* and anaerobic 2.199 ± 0.8702 log_10_ CFU, median 2.1000, *p> 0.9999*) compared to the positive control (0.2% CHX) as shown in **Figures 3** and **4** after 2h and 72h, respectively.

### 3.3 *In vitro* versus *in situ* effects of mouthrinses

**Figure 5:**
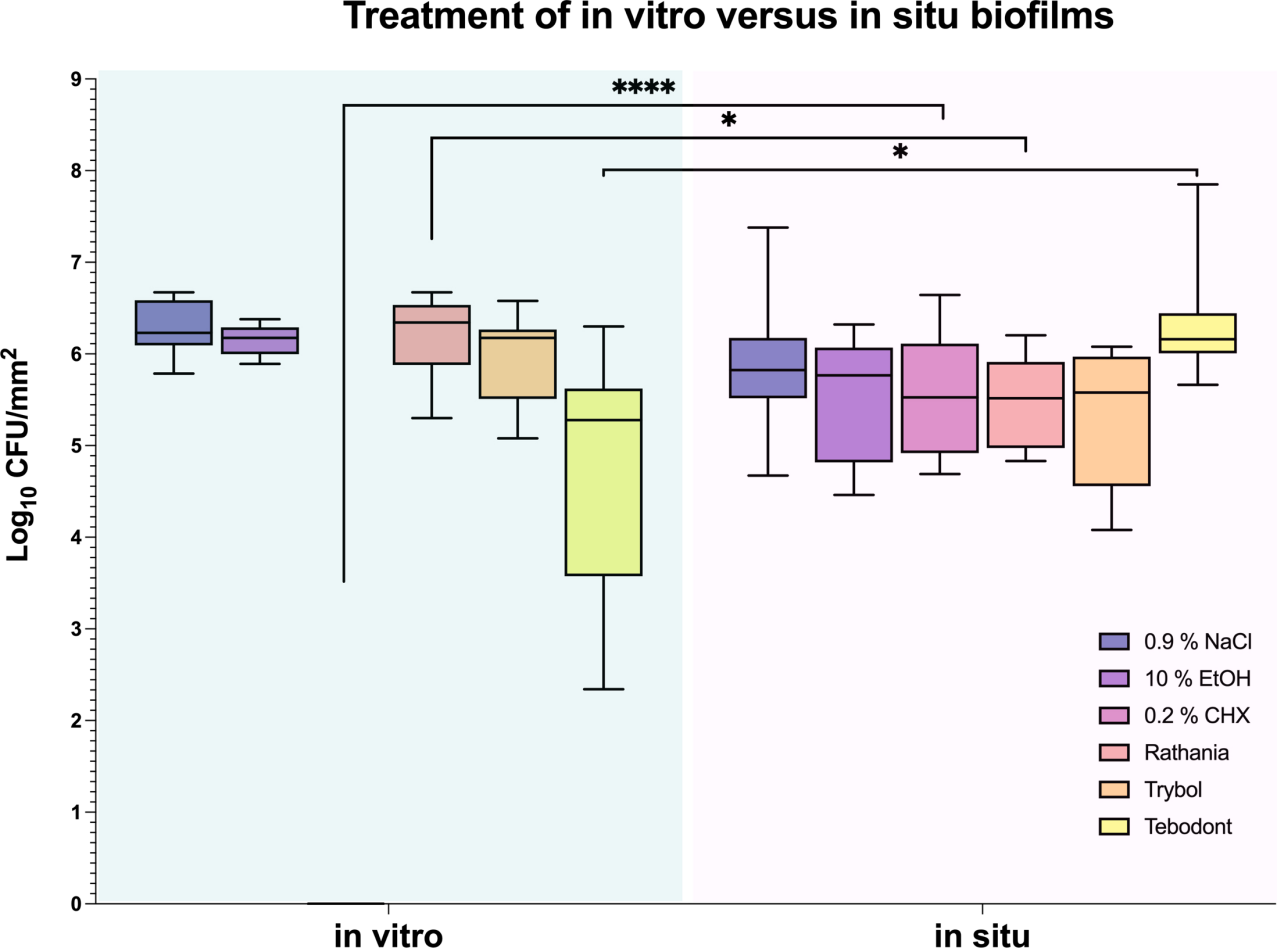
Box plots illustrating the comparison between the two treatment methods *in vitro* and *in situ*. It was measured by colony-forming units (CFUs) on a log_10_ scale per mm^2^ (log_10_/mm^2^). *In vitro* and *in situ* biofilms were treated with Ratanhia, Trybol, Tebodont. 0.2% CHX (positive control), 0.9% NaCl and 10% EtOH (negative control) were also used. The line dividing the box shows the median. The p-values of the significantly different data are plotted: *= P-value <0.05, **** = P-value < 0.0001.

An interesting difference between *in vitro* and *in situ* biofilms (72 h) in regard to the antimicrobial effects of the tested herbal mouthrinses is demonstrated in **Figure 5**. Interestingly, when comparing *in vitro* versus *in situ*, a significant CFU reduction was shown after treatment of the *in vitro* biofilms with 0.2% CHX (0.000 ± 0.000 log_10_ CFU, median 0.000, *p< 0.0001*) or Tebodont (4.708 ± 1.294 log_10_ CFU, median 5.279, *p< 0.0253*). Yet, no significant reduction of the microbial growth for both aerobic and anaerobic microorganisms was found after treatment of the *in situ* biofilms (72h) with 0.2% CHX (5.525 ± 0.645 log_10_ CFU, median 5.525, *p< 0.0001*) or Tebodont (6.400 ± 0.708 log_10_ CFU, median 6.161, *p< 0.0253*). Interestingly, treatment with the mouthrinse Ratanhia led to a less substantial microbial growth (aerobic, anaerobic) *in situ* after 72 h (5.485 ± 0.491 log_10_ CFU, median 5.517, *p < 0.0157*) when compared with the *in vitro* microbial growth (6.208 ± 0.454 log_10_ CFU, median 6.342, *p< 0.0157*). However, the mouthrinse Trybol yielded no significant reduction in both aerobic and anaerobic microbial growth both after 72 h *in situ* (5.331 ± 0.735 log_10_ CFU, median 5.579, *p> 0.1587*) and *in vitro* (5.971 ± 0.498 log_10_ CFU, median 6.176, *p> 0.1587*).

### 3.4 DNA staining and extracellular matrix

**Figure 6:**
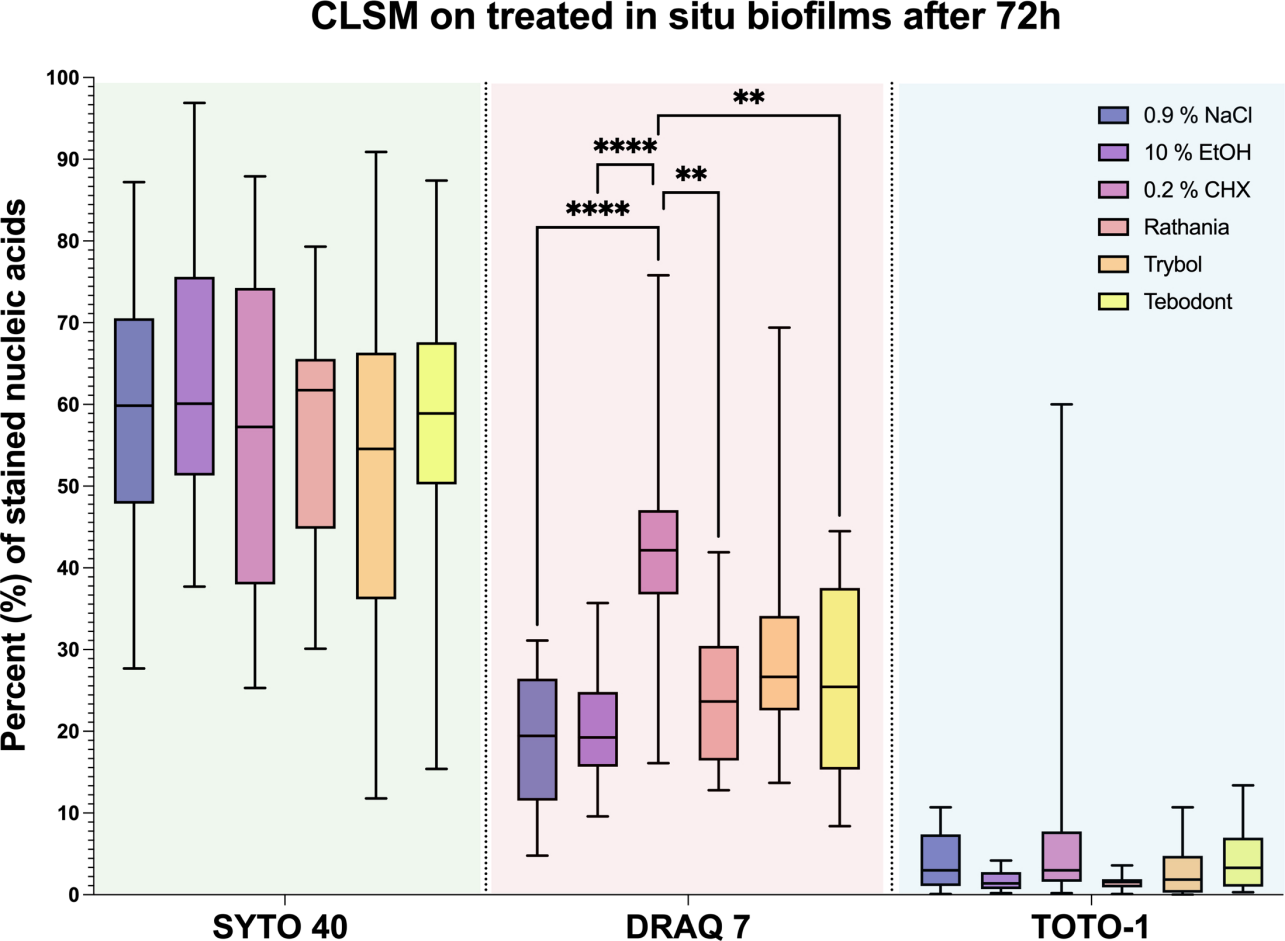
Boxplots illustrating the outcomes of image analysis. The *in situ* biofilm was grown 72 hours on HA discs and afterwards treated with different mouthrinses: 0.2% CHX, 10% EtOH, 0.9% NaCl, Ratanhia, Tebodont and Trybol. The visualization of nucleic acids was aided by SYTO 40, a cell-permeant nucleic acid stain, as well as DRAQ7 and TOTO-1, which cell impermeable DNA dyes. The percentage of stained nucleic acids was measured. The line dividing the box shows the median. The p-values of the significantly different data are plotted: ** = P-value < 0.01., **** = P-value < 0.0001.

**Figure 7:**
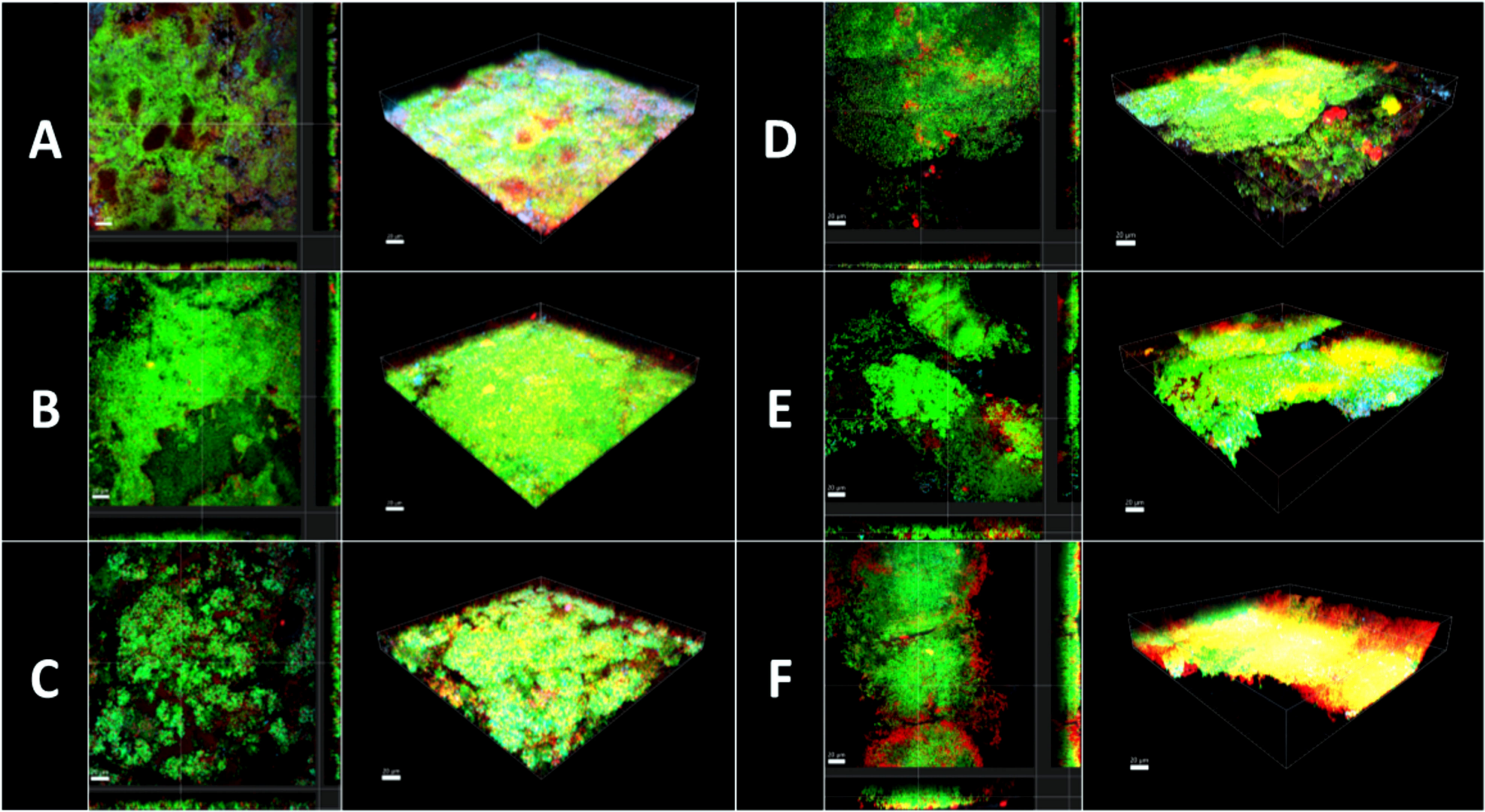
Confocal laser scanning microscopy (CLSM). 3D reconstructions and images sections of biofilm grown 3 days on HA discs and afterwards treated with different mouthrinse **(A)** 0.2% CHX, **(B)** 10% EtOH, **(C)** 0.9% NaCl, **(D)** Ratanhia, **(E)** Tebodont, **(F)** Trybol. The biofilms were used for viability analysis and visualization of extracellular DNA (eDNA): SYTO 40 a cell-permeant nucleic acid stain in green, DRAQ7 in red and TOTO-1 cell impermeable DNA dyes in blue.

The quantification of stained nucleic acids following CLSM microscopy is shown in **Figure 6**, while **Figure 7** demonstrates representative CLSM images after staining of extracellular DNA (eDNA). Prior to the viability analysis and CLSM visualization, the biofilms had been treated with the natural mouthrinses Ratanhia, Trybol, Tebodont, as well as 0.2% CHX (positive control), 10% ethanol and 0.9% NaCl (negative controls). The biofilms were afterwards stained with SYTO 40, a cell-permeant nucleic acid stain in green, which can penetrate intact cells. On the other side, DRAQ7 (red) and TOTO-1 (blue) are cell impermeable DNA dyes. DRAQ7 was used to analyze the inactive (dead) bacteria within the biofilm. Nucleic acids within the extracellular matrix were stained blue by TOTO-1. Although the percentage of stained nucleic acids varied for SYTO 40, no significant impact was observed following the treatment with the herbal mouthrinses. A similar diversity especially for 0.2% CHX is illustrated for TOTO-1 staining. Quantification of the DRAQ7-stained cells revealed that treatment with 0.2% CHX (42.006 ± 12.173 log_10_ CFU, median 42.150) significantly increased the proportions of the dead cells compared with the negative control groups 0.9% NaCl (18.878 ± 8.237 log_10_ CFU, median 19.450, *p> 0.0001*) and 10% ethanol (20.544 ± 6.882 log_10_ CFU, median 19.250, *p> 0.0001*).

Treatment with the herbal mouthrinses as Ratanhia (24.789 ± 8.914 log_10_ CFU, median 23.650, *p< 0.0015*) and Tebodont (26.711 ± 11.566 log_10_ CFU, median 25.450, *p< 0.0073*) yielded higher proportions of vital microorganisms compared to CHX (42.006 ± 12.173 log_10_ CFU, median 42.150). The mouthrinse Trybol yielded for both SYTO 40 and DRAQ7 a high variance in the percentages of stained nucleic acids. Yet, when Trybol was compared with both SYTO 40 (52.983 ± 23.271 log_10_ CFU, median 54.550, *p< 0.9123*) and DRAQ7 (30.150 ± 14.555 log_10_ CFU, median 26.650, *p< 0.1076*) compared to the negative control (0.9% NaCl) there is no statistically significant difference.

Illustrative series of confocal laser scanning microscopy (CLSM) images of biofilms after antimicrobial treatment are shown in **Figure 7**. The panels in **Figure 7** indicated with A-F are images in sections of *in situ* biofilms after 72 h and 3D reconstructions in maximal Z-projections. The panels show the viable microorganisms in green due to staining with SYTO 40, while nucleic acid of dead microbial cells appears in red due to staining with DRAQ 7. Nucleic acids within the extracellular matrix were stained blue by TOTO-1. Panel A represents biofilms treated with 0.2% CHX and clearly shows a higher percentage of red- and blue-stained nucleic acids than panels B and C, which demonstrate the negative controls. Treatment with 0.2% CHX resulted in less dense biofilms compared to treatment with 10% ethanol. Panels B, C, D show only a few microbial cells in red and blue. A fact that points out that treatment with the herbal mouthrinses like Panels B, C, D were less effective than the treatment with 0.2% CHX.

## 4 Discussion

Nowadays, the use of a toothbrush and fluoridated toothpaste is essential when oral hygiene is practiced. However, complete plaque removal is rather unrealistic, so a chemotherapeutic approach could be beneficial in some cases, in which antimicrobial control is mandatory (Zhang et al., 2019). The present study compares the effectiveness of diverse herbal mouthrinses on *in vitro* biofilms versus *in situ* biofilms. This allowed a better understanding of the differences in the outcomes between *in vitro* and *in situ* biofilms after treatment with plant-derived antimicrobial agents.

*In vitro* studies are necessary to evaluate the effectiveness of specific antimicrobial agents and to collect useful information for further *in situ* studies (Charles et al., 2004). For example, an excellent killing effect of 0.2% CHX was shown after the *in vitro* assays, since CHX was able to eradicate all six supragingival species of the *in vitro* biofilm after 64 h. To date, this excellent *in vitro* effect of CHX has been revealed in various reports (Guggenheim and Meier, 2011; Gränicher et al., 2021; Zhang et al., 2021). Interestingly, only Tebodont yielded a significant reduction of the *in vitro* biofilm growth compared to the negative controls. Tebodont yielded a significant reduction of *in vitro* bacterial growth of *A. oris*, *V. dispar*, *F. nucleatum*, *S. mutans* and *S. oralis*. Only for C. albicans there was no significant reduction detected *in vitro* after treatment with Tebodont. The same result was supported by another *in vitro* study, which also confirmed that Tebodont failed to eliminate *C. albicans*. An explanation could be that the strains of *C. albicans* might have been subjected to antifungal activity and become highly resistant to environmental stress (Francisconi et al., 2020). Although a significant reduction in oral microorganisms was detected, the antimicrobial effectivity of 0.2% CHX was much lower compared to that of Tebodont Similarly, the study of Shapiro et al., 1994 supported that sage and tea tree oil inhibited all laboratory strains used in the Zurich biofilm model (Shapiro et al., 1994). In *in vitro* studies it was shown that essential oils with low concentrations can disable enzymes, while essential oils in higher concentrations penetrate the biofilm, rupture the cell wall and thereby eliminate a wide range of microorganisms (Stoeken et al., 2007).

In the present *in situ* study, treatment with 0.2% CHX showed a significant impact against early biofilms after 2 h. Yet, there was no effect against biofilms after 72h compared to the negative controls. Interestingly, the essential oil Trybol achieved a significant outcome *in situ* compared to Tebodont. To confirm this, several clinical studies also concluded that a combination of brushing and rinsing with essential oil containing phenol, thymol, eucalyptol significantly reduced dental plaque and therefore gingivitis compared to only brushing (Stoeken et al., 2007). However, various earlier studies revealed that CHX allowed for a more efficient control of dental plaque compared to mouthrinses with essential oils (Charles et al., 2004; Marchetti et al., 2011). It is surprising that the addition of plant extracts such as ratanhia, myrrh, chamomile, arnica, or sage can yield substantial antimicrobial, anti-inflammatory or epithelium-protecting properties (Shapiro et al., 1994). Especially mouthrinses containing chamomile flower extract contain a wide variety of active chemical nutrients and efficiently reduce dental plaque and gingivitis (Chen et al., 2014). Interestingly, flavonoids, including apigenin, chamazulene and alpha-bisabolol act as anti-inflammatory agents. Herbal ingredients such as sage, myrrh can displace the pH of saliva into the alkaline range and are therefore extremely effective against periodontal diseases (Willershausen et al., 1991). This is in accordance with a recent study which has shown that plant extracts can suppress the supragingival pathogens and thereby reduce plaque accumulation (Bosma et al., 2022). Another widely discussed ingredient of mouthwashes is ethanol, which is commonly used as a solvent in herbal mouthrinses. Ethanol at a high concentration of 40% can interfere with the growth of oral microorganisms, therefore it can be normally found at a concentration 5-27% in different mouthrinses (Brecx et al., 2003). Surprisingly, the mouthrinse Ratanhia contains 57.6% ethanol which when not correctly solved can reduce microorganisms, but can also have some side effects in oral cavity, notably a possible risk for oral cancer, xerostomia or mouth burning (Tiemann et al., 2007; Chen et al., 2014). In the present report, Ratanhia was solved to a final ethanol concentration of 10%. This may have led to non-beneficial outcome as expected for a natural mouthrinse, as the other active ingredients of the mouthwash were also diluted. A comparable outcome was also seen for the other tested mouthrinses, namely Trybol and Tebodont, which yielded a high variation of microbial growth both *in vitro* and *in situ*. One explanation could be that active bacterial communities are heterogenous, dynamic systems that are influenced by multiple internal and external factors, which constantly affect the viability process in the framework of homeostasis. Another fact is that there are individual differences in the microbial counts among the samples and probands which can also lead to high standard deviations (Kurz et al., 2021).

The main aim of our report was to demonstrate the differences between *in vitro* and *in situ* biofilms as study models. The *in vitro* supragingival multispecies “Zurich biofilm model” has been successfully applied for over 25 years for testing the effect of antimicrobial agents in the field of oral medicine (Guggenheim et al., 2004; Ten Cate, 2006). Nevertheless, modern molecular biological investigations have identified over 700 different bacterial species in oral biofilm. The bacteria in their extracellular polysaccharide matrix communicate through signaling molecules, and use an quorum-sensing system to enable their survival (Karygianni et al., 2020; Eick, 2021). These bacteria mainly live under nutrient limitation and are often in a dormant state. Such dormant bacteria react differently to antimicrobial agents than the bacteria that are in a metabolically active state. Furthermore, it has been found that many mouthrinses bind to the extracellular polysaccharide matrix initially before they even reach the bacteria in the deeper layers of the biofilm (Karygianni et al., 2020). Interestingly, in the present study, 0.2% CHX and tea tree oil (Tebodont) showed a statistically significant eradication of supragingival strains of *in vitro* biofilms, but there was no reduction of *in situ* biofilms after 2h and 72h. These findings highlight the reason why 0.2% CHX as well as mouthwashes like Tebodont and Trybol are less effective *in situ* than *in vitro.* Interestingly, solely Ratanhia showed better inhibitory effects *in situ* than *in vitro*. The *in vitro* biofilm model allows for the standardized control of the antimicrobial potential of the mouthrinses, but does not adequately reflect the physiological intra-oral situation (Auschill et al., 2004).

The live/dead viability assay it enabled the visualization of the active and non-active cells of the initial and mature oral biofilm (Kurz et al., 2021). In order to better understand the metabolic process or the turnover rate of saliva it is necessary to choose biofilms which grow directly in the oral cavity and whose three-dimensional structure is not manipulated. Through the use of CSLM, the biofilm structure, thickness and the viability was analyzed *in situ* (Lukic et al., 2020; Sousa et al., 2022). In the present study, it was shown in **Figure 7** that treatment with 0.2% CHX yielded a less dense biofilm compared with the negative control, even though there was no statistical antimicrobial effect. Extracellular DNA (eDNA) is of great importance for microbial adhesion in the early phase of biofilm formation. Thus, the desire to highlight eDNA’s role in biofilm formation has encouraged the visualization of eDNA in the biofilm using the stain TOTO-1 (Schlafer and Meyer, 2017). The CLSM images revealed a wide range of stained nucleic acids (SYTO 40). The percentages of vital microorganisms declined significantly after the treatment with 0.2% CHX. The essential oil Trybol yielded for both SYTO 40 and DRAQ7 a high variability in the percentages of stained nucleic acids. The difference between the vital staining and the CFU values can be attributed to the fact that the microbial aggregates were vortexed and thus, falsify the results (Al-Ahmad et al., 2008). Various studies have proven the advantages of CHX to substantially reduce microbial vitality in oral cavity (Al-Ahmad et al., 2013; Quintas et al., 2015; Kensche et al., 2017). A systematic review showed still a beneficial antibiofilm behavior of different natural ingredients stating that that there is a positive correlation between therapy protocols based on the use of medicinal herbs and the eradication rates of the treated oral biofilm (Karygianni et al., 2015). However, in an earlier study, it was shown that the effectiveness of mouthrinses differed between *in vitro* and *in situ* studies depending on the treatment duration and repeated use of the antimicrobial agent (Auschill et al., 2005). These findings apply to our study, in which the biofilms were only treated once for 2 minutes with the different mouthrinses. The same data confirmed also an earlier study of Arweiler et al., 2004 with a less substantial effect of biofilm density reduction (Arweiler et al., 2004). Furthermore, if fluorescein is applied for a longer time than indicated, microorganisms will not survive and the staining DRAQ7 and TOTO-1 will penetrate the cells, a fact which could lead to a falsification of the results. The major limitation of the study are associated with the visualization of biofilms using CLSM and the subsequent image analysis. The visualization of representative areas on the enamel slabs, the self-fluorescence of the enamel and technical difficulties during quantification were the main challenges of the present study.

In conclusion, natural mouthrinses and 0.2% CHX have a less pronounced effect on *in situ* biofilms, than on *in vitro* biofilms. Both *in vitro* and *in situ* models used in these studies highlighted the efficacy of mouthrinses under short-term exposure, which can lead to different results compared to long-term exposure. More studies are required to clarify the biological mechanisms that contribute to the effect of herbal mouthrinses.

## Data availability statement

The original contributions presented in the study are included in the article/supplementary material. Further inquiries can be directed to the corresponding author.

## Ethics statement

The studies involving human participants were reviewed and approved by the Ethics Committee of the University of Zurich (Basec-Number 2019-01324). Written informed consent for participation was not required for this study in accordance with the national legislation and the institutional requirements.

## Author contributions

NS conducted the experiments, analyzed the data, and wrote this manuscript. PP wrote the application for the Ethics Committee. PP and TA critically reviewed the manuscript. LK and TT conceived the idea for this manuscript, were involved in the data analysis and critically reviewed the manuscript. All authors contributed to the article and approved the submitted version.
